# Sugammadex or Neostigmine for prevention of post-operative pulmonary complications after major abdominal or thoracic surgery: study protocol for the SINFONIA (Sugammadex for preventioN oF pOst-operative pulmonary complIcAtions) randomised controlled superiority trial

**DOI:** 10.1186/s13063-025-08987-4

**Published:** 2025-08-28

**Authors:** Jonathan A. Silversides, Louise Savic, Louise Hiller, Amy Hopkins, Katie Booth, Jennifer Dorey, Richard Smithson, James Mason, Samuel Frempong, Christina May, Ramani Moonesinghe, Ciara M. O’Donnell, Benedict Creagh-Brown, Rebecca Kandiyali, Joyce Yeung, Rupert Pearse, for the SINFONIA investigators, Kirsten Harris, Hannah McNeill, David Vass, Emma Padfield, Dalbir Kaur

**Affiliations:** 1 Wellcome-Wolfson Institute for Experimental Medicine, Queen’s University Belfast, Belfast, UK; 2 Division of Anaesthesia, Theatres and Critical Care, Belfast Health and Social Care Trust, Belfast, UK; 3 Leeds Teaching Hospitals National Health Service Trust, Leeds, UK; 4 Warwick Clinical Trials Unit, University of Warwick, Coventry, UK; 5 Patient and Public Involvement Representative, London, UK; 6 Patient and Public Involvement Representative, Belfast, UK; 7 University College London, London, UK; 8 University of Surrey, Guildford, UK; 9 Queen Mary University of London, London, UK

**Keywords:** Sugammadex, Neostigmine, Neuromuscular blockade, Neuromuscular blocking agents, Respiratory insufficiency, Pneumonia, Acute respiratory distress syndrome, Postoperative complications, Clinical trial

## Abstract

**Background:**

Post-operative pulmonary complications (PPCs) are an important source of morbidity and mortality after major abdominal and thoracic surgery. The use of neuromuscular blocking drugs in general anaesthesia is an important risk factor for PPCs. The incomplete reversal of this neuromuscular blockade at the end of surgery leads to residual weakness of respiratory muscles and predisposes to aspiration of pharyngeal contents, hypoventilation, and thus to PPCs such as atelectasis and pneumonia. Two reversal drugs for neuromuscular blocking agents are available: neostigmine and sugammadex. Compared with neostigmine, sugammadex use results in more rapid reversal of neuromuscular blockade, and small clinical efficacy studies have suggested an associated lower incidence of PPCs. The comparative clinical effectiveness of the two drugs in reducing length of hospital stay or mortality is uncertain. Moreover, a potential safety concern with sugammadex is the relatively high incidence of life-threatening allergic reactions in countries where this drug has been widely used over the last decade.

**Methods:**

SINFONIA is a pragmatic, randomised, open-label, parallel group, superiority trial with an internal pilot which aims to compare the clinical and cost effectiveness of the two available drugs for reversal of neuromuscular blockade, sugammadex and neostigmine, in patients aged 50 years or older undergoing major abdominal or non-cardiac thoracic surgery. The trial will randomise 2500 patients from approximately 40 centres in the UK. The primary outcome will be days alive and out of hospital at 30 days (DAH-30), with key secondary outcomes of PPC incidence, quality of life, and mortality up to 180 days. An embedded observational study will investigate the rate of allergic sensitisation following exposure to sugammadex.

**Discussion:**

The SINFONIA trial addresses an important question for anaesthetists and for patients undergoing major abdominal and thoracic surgery. The choice of reversal agent for neuromuscular blockade between sugammadex and neostigmine is currently largely a matter of anaesthetist preference. A growing body of evidence suggests that sugammadex may reduce the incidence of post-operative pulmonary complications relative to neostigmine. This pragmatic clinical effectiveness trial will provide robust evidence as to the effects of the two drugs on patient-centred outcomes such as DAH-30, as well as on cost effectiveness and the incidence of allergic sensitisation.

**Trial registration:**

The trial was registered on the ISRCTN database (https://www.isrctn.com) prior to opening to recruitment (registration no 15109717).

**Supplementary Information:**

The online version contains supplementary material available at 10.1186/s13063-025-08987-4.

## Administrative information

Note: the numbers in curly brackets in this protocol refer to SPIRIT checklist item numbers. The order of the items has been modified to group similar items (see http://www.equator-network.org/reporting-guidelines/spirit-2013-statement-defining-standard-protocol-items-for-clinical-trials/).

**Table:** 

Title {1}	Sugammadex or Neostigmine for prevention of post-operative pulmonary complications after major abdominal or thoracic surgery: study protocol for the SINFONIA (Sugammadex for preventioN oF pOst-operative pulmonary complIcAtions) randomised controlled superiority trial
Trial registration {2a and 2b}.	ISRCTN – (International Standard Randomised Controlled Trial Number)—15,109,717
Protocol version {3}	V6.0; 30th April 2025
Funding {4}	This project is funded by the National Institute for Health and Care Research (NIHR) Health Technology Assessment Programme (Ref NIHR133056). Additional support for ancillary studies (process evaluation, biological sampling) is provided by the Belfast Health and Social Care Trust.
Author details {5a}	Dr Jonathan Silversides – Queens University Belfast Dr Louise Savic – Leeds Teaching Hospitals NHS Trust Dr Louise Hiller – University of Warwick Mrs Amy Hopkins—University of Warwick Ms Katie Booth—University of Warwick Mrs Jennifer Dorey – Patient/Public Representative Dr Richard Smithson – Patient/Public Representative Prof James Mason—University of Warwick Dr Samuel Frempong – University of Warwick Ms Christina May—University of Warwick Prof. Ramani Moonesinghe – University College London Dr Ciara O’Donnell—Belfast Health and Social Care Trust Dr Ben Creagh-Brown – University of Surrey Dr Rebecca Kandiyali—University of Warwick Prof. Joyce Yeung – University of Warwick Prof. Rupert Pearse – Queen Mary University of London
Name and contact information for the trial sponsor {5b}	Ms Alison Murphy Research and Development Manager Belfast Health and Social Care Trust Tel: 02896 156,057 Email: alison.murphy@belfasttrust.hscni.net
Role of sponsor {5c}	The Belfast Health and Social Care Trust (BHSCT) will act as Sponsor for the trial and the Chief Investigator (CI) will take overall responsibility for the conduct of the trial. The funders have no role in the study design, data acquisition, data analysis or manuscript preparation.

## Introduction

### Background and rationale {6a}

#### Epidemiology and impact of post-operative pulmonary complications

Each year, more than 3 million patients receive a general anaesthetic for a surgical procedure in the United Kingdom (UK) [1]. Complications after surgery and anaesthesia are common, leading to delayed discharge from hospital, higher risk of death, and poor long-term health and quality of life [2]. Post-operative pulmonary complications (PPCs) are the most common complications after surgery, affecting more than 230,000 patients each year in the UK, and have a considerable detrimental effect on patient recovery, survival, and length of hospital stay after surgery [2–5], leading to increased resource use and treatment costs estimated at £280 million per year. While many risk factors for PPCs relate to the surgical procedure or to the patient and are not easily modifiable, the use of Neuro-Muscular Blocking Agents (NMBAs) and their reversal agents as part of general anaesthesia is an important modifiable risk factor [6].

#### Neuromuscular blocking agents and post-operative pulmonary complications

NMBAs are used in general anaesthesia to paralyse skeletal muscle, including the respiratory muscles, to facilitate endotracheal intubation, invasive ventilation, and surgical access. NMBAs have long been recognised as a key risk factor for PPCs [6]. Despite the use of NMBA antagonists to reverse paralysis at the end of surgery, and many safety precautions, residual muscle paralysis still affects a third of patients after surgery [7, 8]. This leaves the patient with weak upper airway and respiratory muscles leading to aspiration of oropharyngeal secretions and reduced hypoxic drive and hypoventilation [9], predisposing to PPCs including pulmonary atelectasis, pneumonia, and respiratory failure [2], which in turn are associated with increased post-operative mortality [4].

#### Reversal of neuromuscular blocking agents (NMBAs)

Anaesthetists choose between two drugs to reverse the effects of NMBAs, the anti-cholinesterase drug neostigmine and the NMBA binding drug sugammadex. Neostigmine is itself associated with dose-dependent muscle weakness and impaired lung function [10]. Neostigmine also causes severe bradycardia necessitating co-administration of an anti-cholinergic drug such as glycopyrrolate or atropine, which have autonomic side effects. Compared to neostigmine, sugammadex reverses NMBAs more rapidly and reliably, reducing the incidence of residual muscle weakness after surgery [11].

#### Choice of NMBA reversal agent and post-operative pulmonary complications

A Cochrane systematic review of 41 clinical trials of 4206 patients compared the efficacy and safety of sugammadex with neostigmine for NMBA reversal. Sugammadex use was associated with lower rates of post-operative residual paralysis and fewer hypoxic events after surgery [11]. In a propensity-matched study of 45,712 patients undergoing surgery with NMBAs in 12 US hospitals, sugammadex was associated with a reduced risk of pneumonia and respiratory failure (OR 0.70, [95% confidence interval 0.63–0.77]) compared to neostigmine [12]. Several smaller observational studies and randomised trials have also identified an association between sugammadex use and a reduced incidence of PPCs [13–18]. A recent systematic review and meta-analysis (43 trials, 5839 participants) found sugammadex to be associated with lower rates of PPCs compared with neostigmine (16 trials, *N* = 1570, OR 0.67 (95% CI 0.47–0.95)), but uncertain effects on patient-centred outcomes (days alive and out of hospital at 30 days (DAH-30), length of stay, mortality) [19].

#### Sugammadex and allergy

Anaphylaxis complicates up to one in 2500 anaesthetics and is associated with a 10% mortality rate as well as long-term sequelae for survivors [20]. For the SINFONIA trial population, one in ten patients die following these reactions, while survivors commonly experience long-term complications such as kidney injury, cognitive impairment, and post-traumatic stress disorder [20]. Life-threatening anaphylactic reactions to sugammadex are currently rare in the UK, with only one case among 64,000 doses administered [21]. By contrast, in Japan where sugammadex is used in 95% of anaesthetics, repeat exposure is common and sugammadex has become the most frequent cause of perioperative anaphylaxis with an incidence of one in 5000 administrations [22, 23]. If sugammadex becomes the NMBA reversal drug of choice in the UK National Health Service (NHS), this could lead to a significant increase in perioperative anaphylaxis over the next decade [24]. In comparison, neostigmine has an allergy risk approaching zero, with only one reported case in the last 20 years in the UK [25].

The potential rate of sensitisation to sugammadex following a single exposure can be estimated using standard NHS allergy tests. Sensitisation is demonstrated by the presence of IgE antibodies, as detected through skin tests (skin prick and intra-dermal testing) and mast cell activation testing (which requires a blood sample). Sensitisation indicates a significantly increased risk of allergic reactions during future exposure to the drug, but will not provide a precise risk estimate, since many participants with antibodies will not experience a clinical allergy on re-exposure. The true significance of the sensitisation, and consequent risk of anaphylaxis, can be estimated by administering the drug in small doses in a controlled environment (challenge test).

## Hypothesis and objectives {7}

We hypothesise that in adults aged 50 years or older, the use of sugammadex for reversal of neuromuscular blockade at the end of elective or emergency major abdominal or non-cardiac thoracic surgery will be associated with fewer PPCs, and thereby a greater number of days alive and out of hospital at 30 days compared with neostigmine.

The primary objective of SINFONIA is to determine whether sugammadex is superior to neostigmine after elective or emergency major abdominal or non-cardiac thoracic surgery in terms of days alive and out of hospital at 30 days (DAH-30).

Secondary objectives are to determine whether sugammadex is superior to neostigmine in terms of prevention of PPCs, mortality and other patient-centred outcomes after elective or emergency major abdominal or non-cardiac thoracic surgery; to determine the cost effectiveness of sugammadex compared with neostigmine; and to estimate the rate of allergic sensitisation after a single exposure to sugammadex.

## Trial design {8}

SINFONIA is an open-label, pragmatic, randomised (1:1) superiority trial with an internal pilot which aims to compare the clinical and cost-effectiveness of two drugs, sugammadex and neostigmine for the reversal of neuromuscular blockade in a high-risk population.

The 12-month internal pilot phase was intended to assess feasibility, as evidenced by site opening and recruitment. This was completed successfully on 31 st January 2024. The trial continued seamlessly into the main phase of recruitment.

An embedded observational substudy will investigate the incidence of allergic sensitisation after exposure to these drugs. Combining the results of allergy testing and incidence of clinical hypersensitivity reactions to sugammadex during the trial, and comparing these with current best estimates of positive allergy testing to other drugs used in the perioperative period and incidence of clinical hypersensitivity to these, as well as the incidence of re-exposure to sugammadex, we aim to estimate the likely incidence of sugammadex hypersensitivity reactions as usage increases across the UK. We anticipate that future longitudinal studies of sugammadex hypersensitivity will strengthen these findings.

## Methods: participants, interventions, and outcomes

### Study setting {9}

The trial will take place in approximately 40 National Health Service hospitals across the United Kingdom. A full list of study sites is available on request. Approximately five of these centres will participate in the embedded allergy sensitisation sub-study.

### Eligibility criteria {10}

Inclusion criteria:

1. Patients presenting for elective or emergency major abdominal or non-cardiac thoracic surgery*
2. Age ≥ 50 years
3. Planned use of rocuronium or vecuronium for neuromuscular blockade
4. Planned reversal of neuromuscular blockade at the end of surgery

*See Additional File 1 for examples of eligible major surgical procedures

Exclusion criteria:

1. Known allergy to sugammadex, neostigmine, or glycopyrrolate.
2. Lack of written informed consent for trial participation.
3. Planned invasive mechanical ventilation before or after surgery.
4. Previous participation in SINFONIA trial.
5. Clinician refusal (with reason).

### Who will take informed consent? {26a}

Potentially eligible participants are identified through multidisciplinary meetings, pre-operative assessment clinics and/or operating theatre lists at participating hospitals and screened against inclusion/exclusion criteria (Table 1). The site principal investigator (PI) or medically qualified delegate will confirm eligibility.

**Table 1:** Participant timeline {13}

Visit	0	1	2	3	4	5	6
Visit window	Baseline (pre-op)	Day 0 (surgery)	Day 1	Day 7	Day 30	6 weeks to 6 months	180 days
Screening using inclusion/exclusion criteria	X						
Informed consent	X						
Enrolment	X						
Baseline data	X						
Randomisation		X					
Intervention		X					
PPCs				X*			
Duration of hospital stay					X		
Duration of intensive care stay (if applicable)					X		
Survival status					X		X^+^
Hospital readmission					X		
Health resource use					X^#^		X
Quality of recovery (QoR-15)			X				
Health-related quality of life (EQ-5D-5L)	X			X^#^	X^#^		X^^^
~ *Skin testing for allergic sensitisation*						X	
~ *Blood sampling for mast cell activation test*		X				X	
^*@*^*Blood sample collection for future use*	X^@^		X^#^				

^*^Or until hospital discharge if sooner; ^#^Or as close as possible; ^^^ ± 28 days

~ *Allergic sensitisation substudy participants only*

^*@*^*Selected patients only. Baseline sampling at any point between consent and surgical incision*

^+^Survival check will also be done prior to the dissemination of trial results at the end of trial

Potential participants are approached at the first suitable opportunity by a member of the research team, e.g. principal investigator or research coordinator, by telephone, online, or face-to-face consultations. A patient information leaflet is provided, and after appropriate opportunity for consideration, written informed consent may be obtained. Potential participants who lack capacity to give or withhold informed consent will not be recruited. If a participant loses capacity during their participation in the trial, the original consent by the participant will be respected. Details of patients who are potentially eligible for the trial but not recruited will be recorded.

### Additional consent provisions for collection and use of participant data and biological specimens {26b}

Patient identifiable data will not be entered into the trial database. However, consent will be obtained for the SINFONIA trial team to review patient data for the purposes of monitoring and oversight. Consent will be sought for use of non-identifiable data in future studies. Participants will have the opportunity to provide an email or postal address for contact with results at the end of the trial.

Patients at participating sites will have the option to consent for a substudy investigating allergic sensitisation, or to additional blood sampling for future use. Participation in these substudies is not required for participation in the main trial.

## Interventions

### Intervention description {11a}

#### Explanation for the choice of comparators {6b}

At the time of trial inception, the drug which represented the standard of care, used in most cases for reversal of neuromuscular blockade, was neostigmine.

##### Sugammadex

Participants randomised to the sugammadex arm should receive an intravenous bolus of sugammadex (2–4 mg/kg) for reversal of neuromuscular blockade around the end of the surgery. Within these parameters, the precise dose and timing are left to the discretion of the treating anaesthetist. If deemed necessary by the treating anaesthetist, patients allocated to the sugammadex treatment group may be administered a second dose of sugammadex. The maximum total dose of sugammadex (whether one or two doses are used) should not exceed 8 mg/kg.

##### Neostigmine

Participants randomised to the neostigmine arm should receive an intravenous bolus of neostigmine (30–70 mcg/kg) for reversal of neuromuscular blockade around the end of surgery, with co-administration of glycopyrrolate at an appropriate dose to prevent muscarinic side effects (for example 200 mcg per 1 mg of neostigmine). The precise dose and timing are left to the discretion of the treating anaesthetist. If deemed necessary by the treating anaesthetist, patients allocated to the neostigmine treatment group may be administered a second dose. The maximum total dose of neostigmine (whether one or two doses are used) should not exceed 5 mg or 70 mcg/kg, whichever is less.

### Criteria for discontinuing or modifying allocated interventions {11b}

N/A. As this trial involves intervention at a single point in time, there will be no opportunity to discontinue or modify the allocated intervention other than as described above.

### Strategies to improve adherence to interventions {11c}

As the intervention occurs at a single timepoint, strategies to improve adherence to interventions are focused on (1) ensuring that the patient is eligible to receive a trial intervention up to the point of delivery, (2) avoiding crossover, and (3) ensuring delivery of protocol-specified drug doses.

#### Eligibility

To be eligible for the trial, patients must receive a neuromuscular blocking agent (rocuronium or vecuronium) which can be reversed with either investigational medicinal product (IMP). Other NMBAs can be used in clinical practice, and this is largely based on anaesthetist preference. The degree of neuromuscular blockade present at the end of surgery depends on drug dose used, individual pharmacokinetic and pharmacodynamic factors, and the timing of doses relative to the duration of surgery. Reversal of neuromuscular blockade using either agent should therefore be guided by neuromuscular monitoring, for which guidelines exist [26–28]. Some anaesthetists may elect not to use a reversal agent if monitoring suggests adequate spontaneous recovery of neuromuscular function. Other possible scenarios include last-minute anaesthetist scheduling changes, operative findings, or an unexpected intra-operative complication such that mechanical ventilation is continued post-operatively in an intensive care unit, in which case a reversal agent would typically not be indicated. Eligibility to receive any IMP is thus dependent on anaesthetist, surgeon and patient factors which are dynamic over the course of a surgical procedure.

#### Crossover

Due to more rapid onset of action with sugammadex than neostigmine, and ability to reverse more profound degrees of neuromuscular blockade, the risk of crossover is greatest from neostigmine to sugammadex. Possible scenarios include the presence of deep neuromuscular blockade at the end of surgery, for example if surgery is completed more quickly than expected or a larger dose of neuromuscular blocking drug is given towards the end of the case.

Strategies to ensure eligibility and avoid crossover therefore include (a) encouraging, though not mandating, that randomisation occurs close to the timepoint when neuromuscular reversal is given, i.e. as late as possible, (b) encouraging clear communication between research delivery teams and treating anaesthetists to ensure eligibility for the trial is confirmed as close as possible to the time of randomisation, and (c) providing written guidance to anaesthetists to remind them of national guidelines supporting the routine use of quantitative neuromuscular monitoring to guide dosing of neuromuscular blocking drugs and reversal agents. These measures are intended to minimise the risk of a trial participant being randomised but not receiving a reversal agent.

#### Dosing

The trial protocol mandates a specific weight-based approach to dosing, and this dosing regimen is conveyed in written or electronic format at the time of randomisation to trial teams and to anaesthetists as well as in the protocol and at site initiation visits. However, this is a pragmatic clinical effectiveness trial, which aims to compare the two interventions in the context of real-world clinical practice, which involves drug dosing being individualised by anaesthetists based on numerous clinical and clinician-specific factors. In this context, at least some of this variation is valid. Following review of data after 1 year, we introduced a protocol amendment to address some of this variation. These actions included (a) calculation and notification of an individual patient dose based on a baseline weight to trial teams and anaesthetists, (b) additional clarification on the circumstances in which a dose outside the protocol-specified range would be considered acceptable, and capture of the justification supplied e.g. based on results of neuromuscular monitoring, or dose adjustment for obese patients on the basis of local/national guidelines, (c) site education and additional information in the form of a dosing table to be distributed to sites, and (d) addition of a process evaluation to investigate the basis for non-compliances.

## Relevant concomitant care permitted or prohibited during the trial {11d}

All patient care outside the SINFONIA trial intervention will be conducted according to routine clinical practice and local guidelines. Anaesthetists will be reminded of best practice with regards to neuromuscular monitoring, in keeping with national guidance [28].

## Provisions for post-trial care {30}

As this is a pragmatic comparative effectiveness trial with an intervention applied at a single time point, post-trial care will be the responsibility of clinical teams with no additional provision from the trial team. Indemnity will be provided through Queen’s University Belfast for the design of the study and through the NHS indemnity scheme for harm to trial participants resulting from study conduct.

## Outcomes {12}

The primary outcome is Days Alive and out of Hospital at 30 days after surgery (DAH-30) [29]. This is a patient-centred composite outcome which encapsulates post-operative complications which impact on hospital length of stay and mortality.

Secondary outcome measures include the following:

1. Clinical effectiveness measures
  - Post-operative Pulmonary Complications (PPCs) [30] up to seven days after surgery, or hospital discharge (whichever is sooner).
  - Mortality rates at 30 and 180 days after surgery.
  - Quality of Recovery (QoR-15) on day 1 after surgery.
  - Health-related quality of life at 7, 30, and 180 days after surgery (EQ-5D-5L)
2. Safety outcome
  - Allergic reactions within 24 h of study drug administration
3. Health economic outcomes
  - Health resource use over 180 days after surgery
  - Quality-adjusted life years at 180 days
4. Other outcomes
  - Rate of allergic sensitisation to sugammadex (in patients enrolled in the allergic sensitisation substudy) identified at allergy follow-up clinic between 6 weeks and 6 months after surgery, defined by:
    i. Positive skin test
    ii. Positive sugammadex challenge test
  - Correlation between mast cell activation testing (a novel diagnostic tool of uncertain clinical utility) and current gold standard diagnostic tools (skin testing and provocation testing)

## Sample size {14}

The trial will include 2500 participants (1250 per trial arm). Based on data from the Secure Anonymised Information Linkage (SAIL) databank in Wales, we expect participants in the neostigmine arm to experience a mean DAH-30 of 22.4 days (SD 7.4). Assuming a standard deviation of 7.5, with a 5% two-sided significance level and 90% power, the randomisation of 2500 participants will allow detection of a one-day difference in DAH-30 between trial arms, whilst allowing for a 5% loss to follow up, e.g. due to withdrawal of consent or transfer to a non-participating hospital. This sample size will also allow detection of a 3% absolute difference in the incidence of PPCs between trial arms with at least 85% power and 5% two-sided significance, assuming a PPC rate of approximately 7%.

The allergic sensitisation substudy will recruit up to 250 participants, 200 of whom will have received sugammadex and 50 neostigmine. No formal sample size calculation was carried out for the substudy.

## Recruitment {15}

This is a pragmatic trial with broad eligibility criteria and a relatively straightforward intervention involving minimal change to usual care. The eligibility criteria are broad, and it is estimated that over 150,000 patients fulfil these criteria each year around the UK. The trial is recruiting across approximately 40 sites and no major difficulties in achieving target recruitment are anticipated.

## Assignment of interventions: allocation

### Sequence generation {16a}

Participants will be randomised on a 1:1 basis to receive either sugammadex or neostigmine. Randomisation will be undertaken through a simple and secure web-based or Interactive Voice Response Randomisation System (IVRS) randomisation system that has been established by the programming team at Warwick Clinical Trials Unit (WCTU). This computerised procedure will use a minimisation algorithm to ensure balance in treatment arm allocation across the following stratification variables, factors thought to affect outcome either through treatment effectiveness or underlying prognosis, also permitting appropriate exploratory subgroup analyses:

1. Trial hospital site
2. Emergency vs elective surgery
3. Thoracic vs abdominal surgery

The inclusion of hospital site within this list of factors allows for some instances of predictability of the next treatment allocation. To eliminate this, each patient will have a probability (unspecified here) of being randomised to the opposite trial arm that they would have otherwise received.

### Concealment mechanism {16b}

The randomisation system at the WCTU will not release the randomisation code until a patient has been recruited into the trial, thus ensuring concealment of allocation.

### Implementation {16c}

Randomisation should be undertaken after induction of anaesthesia where possible, although this is not mandatory. However, it is mandatory that randomisation is completed before neostigmine, sugammadex or any neuromuscular blockade reversal drug is administered.

If the web-based or IVRS enrolment/randomisation system is unavailable for technical reasons, an emergency enrolment/randomisation telephone system will be provided by WCTU.

## Assignment of interventions: blinding

### Who will be blinded {17a}

Care will be taken not to inform participants of treatment group assignment, except where necessary for patient safety (e.g. allergic reaction). Research delivery staff assessing participant outcomes will be masked to treatment group assignment wherever possible. However, complete blinding of all clinicians and research staff is not feasible, and IMP administered will be recorded in the clinical notes according to usual clinical care.

During the trial, the Trial Management Group and the Trial Steering Committee will not see outcome results broken down by treatment arm.

### Procedure for unblinding if needed {17b}

The clinical team will not be blinded to the IMP administered, and this will be recorded in the clinical notes, so there will be no specific unblinding procedure.

## Data collection and management

### Plans for assessment and collection of outcomes {18a}

Baseline, perioperative, and outcome data will be collected by trained site research staff from medical notes using an electronic or paper case report form (CRF). Baseline EQ-5D-5L questionnaires (available from www.euroqol.org) will be completed by face-to-face or telephone interview prior to surgery, or if necessary (e.g. in the case of emergency surgery), by retrospective completion at the earliest opportunity post-operatively. QoR-15 questionnaires [31] will be completed face-to-face by a member of the site research team on post-operative day 1.

Day 30 follow up for hospital readmission and mortality will be completed by review of medical records, and if necessary, by telephone contact by site research staff with the participant or their General Practitioner. Participants will be contacted by telephone and/or email at 30 days post-surgery (or as close as possible) and again at 180 days (or as close as possible) by site research staff. During these contacts, data on health resource use will be collected, and quality of life will be assessed using the EQ-5D-5L questionnaire. If patients are physically unable to complete the EQ-5D-5L questionnaire, this can be completed by a member of the research team or a carer on their behalf.

## Allergic sensitisation substudy

### Baseline blood sampling and processing

Where possible, 10 mL of blood will be taken following induction of anaesthesia and prior to the administration of the IMP (either pre- or post-randomisation). Where this is not possible, the blood sample will be taken as close as possible to IMP administration. The samples will be processed in the hospital laboratory, frozen, and stored. Samples will be transported in batches to the immunology laboratory at Leeds Teaching Hospitals NHS Trust, at a time of mutual convenience, where mast cell activation testing will be performed. Any samples not used will be disposed of in accordance with local policy and applicable regulations.

### Follow-up testing at 6 weeks to 6 months

Following baseline blood sampling, and depending on group allocation, participants will undergo a clinical assessment, comprising review of notes and/or telephone or face-to-face consultation by an allergy specialist. This assessment will determine suitability for skin testing. Participants will be asked to attend a local drug allergy clinic between 6 weeks and 6 months after their surgery for repeat blood sampling (for mast cell activation) and, if appropriate, a skin test. In line with routine drug allergy skin testing clinics, some medications may be discontinued for a period of one week prior to testing. Where appropriate in the clinical judgment of the allergy specialist, skin testing will be performed at an allergy clinic. This comprises skin prick and intradermal testing on the forearm of both arms, each test 2 cm apart. Positive and negative control tests will be performed on the forearm using histamine and saline respectively prior to skin prick testing, to ensure that the patient’s skin is appropriately reactive. A negative control saline intradermal test will be performed prior to intradermal testing, to confirm appropriate skin reactivity. The testing takes around one hour in total and involves minor discomfort from the slight scratching of the skin with each test. Any skin test developing a wheal of 3 mm or greater than the negative control site is usually considered positive, but the result of the test is a clinical judgment of the allergy specialist. Systemic reactions to skin testing are rare but can occur, typically involving urticaria and a sensation of itch, although more severe allergic reactions are possible. The testing will be performed by specialists who are trained and experienced in the conduct and management of skin testing, and who will treat any symptoms which occur in line with local and national guidance. The patient will be contacted between 48 h and one week following testing to check whether any of the test sites developed localised redness or swelling, which indicates a possible delayed-type allergic reaction. In line with standard drug allergy testing procedure, patients may be offered a drug provocation test, whereby they are given small doses of sugammadex in a carefully monitored setting at the earliest practical opportunity. This is both for future safety of the patient and to understand the specificity of skin testing.

## Process evaluation

A process evaluation (PE) will be undertaken within the SINFONIA trial, led by collaborators at Queen’s University Belfast. It is likely that the complex decision-making processes around use and dose selection of neuromuscular blockade reversal agents (NMBAs) in major surgery are influenced by a range of organisational, clinician, and patient-related factors. It is important to develop a detailed understanding of these factors to explore the relationship between intervention delivery and trial outcomes. To understand this complexity and the likely variation in intervention delivery, we plan to embed a pragmatic PE within the trial to explore adherence to the intervention and the influence on patient and trial outcomes. A protocol for this process evaluation will be made available as a separate publication.

## Plans to promote participant retention and complete follow-up {18b}

Follow-up data will be collected for all randomised participants, unless they explicitly request to be withdrawn from the trial. If such a request is received, participants will be asked for consent for data collection from medical records to continue without further contact.

## Data management {19}

### Data collection

We will use a web-based Clinical Data Management System (CDMS) for data management, developed by the WCTU programming team. All specifications (i.e. database variables, validation checks, screens) will be agreed between the programmer and appropriate trial staff. Participant data will be collected in accordance with the protocol. Clinical data will be collected during the hospital stay up to 30 days after surgery, and thereafter from contact with the patients and their clinical teams up to 180 days. The case report forms (CRF) will be developed by the WCTU and made available to the participating sites as paper and electronic CRFs (eCRF) for ease of data collection. The trial will be conducted in accordance with the current approved protocol, Good Clinical Practice (GCP), relevant data protection regulations, the trial Data Management Plan, and standard operating procedures (SOPs). A monitoring plan and risk assessment will be devised to protect participant safety and the integrity of trial data.

### Data checks and validations

Input fields in the CDMS will be assigned validation rules as appropriate to prevent erroneous data being saved back to the database. Forms can only be saved once all input fields pass the validation rule checks. All validation rules will be documented in a Functional Requirement Specification. The trial team will implement processes to conduct manual checks on data entered by participating sites to maximise completion and accuracy of data collected. Data checking processes will be documented according to a trial-specific Data Management Plan which will be developed between the trial team, Chief Investigator, statisticians, health economists, and quality assurance staff.

### Data storage

All essential documentation and trial records will be stored by WCTU in conformance with the applicable regulatory requirements and access to stored information will be restricted to authorised personnel. Any paper data forms will be stored in a lockable filing cabinet in a secure room, to which access is restricted to authorised personnel. Electronic data will be stored in a secure area of the computer with access restricted to staff working on the trial and the WCTU Quality Assurance team. All databases containing identifiable information will be encrypted and password protected. Any data that are transferred out of the secure environment will adhere to Warwick SOPs.

### Data access and quality assurance

All data access will be controlled by individual usernames and passwords, and any changes to data will require the user to enter their username and password as an electronic signature in accordance with regulatory requirements. Staff will have access restricted to the functionality and data that are appropriate for their role in the trial and will not share their log in details.

### Archiving

Trial documentation and data will be archived for at least ten years after completion of the trial. Trial Master File and associated data will be archived by WCTU; trial data generated at sites will be archived for a minimum of 10 years and in accordance with local policy.

## Confidentiality {27}

The trial complies with all applicable UK legislation and WCTU Standard Operating Procedures. All data are being stored securely and held in accordance with the UK General Data Protection Regulations. All CRFs, questionnaires, trial reports, and communication regarding the trial will identify the participants by the assigned unique trial identifier only. Participant confidentiality will be maintained at every stage, and identifiable information will not be made publicly available to the extent permitted by the applicable laws and regulations. Personally identifiable data will not be transferred outside of the participating site other than (a) where contact details (email or postal address) have been voluntarily provided, with consent, by the participant for the purposes of receiving notification of trial findings, or (b) for trial monitoring purposes or compliance with regulatory requirements.

## Plans for collection, laboratory evaluation and storage of biological specimens for genetic or molecular analysis in this trial/future use {33}

In selected study sites, biological samples will be collected which will be stored for future analysis as part of a separate research programme aiming to understand biological phenotypes in the perioperative period. This work will be subject to separate ethical approval and separate funding application(s). With patient consent, 30 mL blood samples (approximately) will be taken on two occasions: at baseline (any time between consent and the beginning of the surgery) and on post-operative day 1 at 12–24 h following the end of surgery or as close as possible. This will be processed according to a specified standard operating procedure. Samples will be transferred in batches, at a time of mutual convenience, to a laboratory at Queen’s University Belfast for analysis and storage for future use. If a patient is enrolled but not randomised, sampling and data collection will proceed unless the patient is specifically withdrawn from the substudy.

## Statistical methods

### Statistical methods for primary and secondary outcomes {20a}

All randomised participants, regardless of whether they received their randomised intervention or not, will be included in all trial analyses according to their randomised allocation using all data collected up to their final follow-up in the trial (180-day time point, or their censor date if withdrawn or lost to follow-up). As the protocol states that randomisation should to be undertaken after induction of anaesthesia, where possible, withdrawal of consent prior to surgery is not anticipated, although later withdrawal from follow-up could occur and such participants will not be included in relevant denominators. Where a participant specifically requests to withdraw from follow-up, data collected up to the point of withdrawal will still be used, and permission will be sought to collect data from medical records without contacting the participant. In this way, all primary statistical analyses will be undertaken on an intention to treat basis where possible to preserve randomisation, avoid bias from exclusions and preserve statistical power.

For the primary outcome of DAH-30, appropriate point estimates and measures of variation will be reported and presented graphically for each of the two randomised arms (as determined by the distribution of the data). Randomised arms will be compared using appropriate regression techniques for count data, determined following examination of the distributions of the trial data and consideration of possible under- or over-dispersion, but anticipated to be zero-inflated Poisson regression model or zero-inflated negative binomial regression model. The model will include minimisation variables (hospital site, emergency vs elective surgery, thoracic vs abdominal surgery).

The secondary outcomes of PPC rates and mortality rates will be assessed across randomised arms using chi-squared tests, with additional logistic regression used to adjust for minimisation variables. Participant’s Quality of Recovery (QoR-15) score will be calculated using the appropriate scoring manual and reported by randomised arm using appropriate point estimates and measures of variation.

The safety outcome of allergic reaction rates and allergic sensitisation substudy findings will be presented using descriptive statistics. A comparison of the risks of different patient groups having 0, 1, 2, or 3 positive results from these tests will be explored using appropriate regression techniques for count data, determined following examination of the frequency of incidences within the trial. The incidence of positive allergy testing to sugammadex will be compared to current estimates of baseline population sensitisation to rocuronium, providing a comparator with which to understand future sensitisation rates to sugammadex.

A Consolidated Standards of Reporting Trials (CONSORT) flow diagram will illustrate the frequency of participants:

- Assessed for eligibility.
- Excluded prior to randomisation (and the frequency of each reason for exclusion).
- Randomised.
- Allocated to each randomisation arm.
- Receiving or not receiving their randomised treatment.
- Followed up at each protocol specified timepoint (and reasons for loss to follow-up).

## Interim analyses {21b}

No interim analyses will be undertaken.

## Methods for additional analyses (e.g. subgroup analyses) {20b}

Pre-specified hypothesis-generating sub-group analyses, using the variables defined within the minimisation algorithm, will be undertaken using appropriate modelling techniques. These exploratory sub-group analyses will have lower power than the main trial analysis and results will be scrutinised graphically via forest plots.

## Methods in analysis to handle protocol non-adherence and any statistical methods to handle missing data {20c}

With the protocol stating that randomisation should occur after induction of anaesthesia, where possible, and the main data collection time window being only 30 days from surgery, missing data due to withdrawals or lost to follow-up are not anticipated. With efforts to collect full baseline, treatment, and follow-up data on all trial participants, it is thus anticipated that missing data will be minimal. Participants with missing primary outcome data will not be included in the primary analysis in the first instance. However, this presents a risk of bias, and thus consideration will be given to any missing data in the form of a sensitivity analysis to determine the degree to which the conclusions may change with different missing data assumptions. If applicable after investigation into the degree of treatment crossovers, a secondary per-protocol analysis may be undertaken for sensitivity purposes, including only participants who received their randomised intervention.

## Health economic evaluation

A prospectively planned cost-effectiveness analysis will be conducted from an NHS and personal social services perspective, following NICE guidelines [32]. Resource use attributable to neuromuscular blockade reversal during the initial hospital stay will be recorded via CRFs, including length of hospital stay. Community healthcare contacts within 6 months post-surgery will be recorded. Healthcare resource use will be costed using the latest published national reference costs, reflated to the most recent year [33]. Resource use will be reported separately for hospital and community services.

Generic health-related quality of life will be measured using the EQ-5D-5L questionnaire at four time points: baseline, 7 days, 30 days, and 6 months post-intervention. The EQ-5D-5L scores will be converted to health status scores using the UK value set recommended by NICE at the time of analysis [34, 35]. QALYs will be calculated for each participant using the area under the curve (AUC) method, employing the trapezoidal rule to interpolate between time points. Baseline EQ-5D-5L scores will be included to minimise bias in QALY calculations and to adjust subsequent analyses [36, 37]. Whilst more frequent measurements would be ideal, the measurement schedule is necessarily pragmatic within the design of the trial.

A within-trial cost-effectiveness analysis (to 6 months) will be conducted using bivariate regression of costs and QALYs, enabling a probabilistic assessment of incremental treatment cost-effectiveness [38]. Mechanisms of missingness of data will be explored and multiple imputation methods will be applied to impute missing data [39–41]. Imputation sets will be used to estimate incremental cost per QALY estimates and confidence intervals. Findings will be analysed and visualised on the cost-effectiveness plane, as cost-effectiveness acceptability curves, net monetary benefit and value of information analysis. Should costs and quality-of-life not converge within 6 months, more extensive economic modelling using decision-analytic methods may be considered to extend the target population, time horizon and decision context, drawing on best available information from the literature and stakeholder consultations to supplement the trial data. Parameter uncertainty in the decision-analytic model will be explored using probabilistic sensitivity analysis. If longer term decision modelling is to be undertaken, then costs and outcomes will be discounted at 3.5% after the first year of randomisation in line with NICE reference case [32]. All analyses and modelling will be performed using Stata 16 SE (or the latest available version). The study’s economic evaluation reporting will adhere to the Consolidated Health Economic Evaluation Reporting Standards (CHEERS) statement [42].

## Plans to give access to the full protocol, participant level-data and statistical code {31c}

The full trial protocol is available at www.warwick.ac.uk/sinfonia. Research groups requesting the trial dataset and statistical code should include a qualified statistician and initially address the chief investigator, providing a full proposal for their use of the data, for review and approval by the SINFONIA trial management group. The data required for the approved, specified purposes will be provided, after completion of a data sharing agreement. Data sharing agreements will be set up by the sponsors of the trial, the funders, the trial coordination centre, and the trial steering and management groups.

## Oversight and monitoring

### Composition of the coordinating centre and trial steering committee {5d}

The trial is coordinated by the University of Warwick Clinical Trials Unit, who will be responsible for day-to-day management of the trial under the supervision of the Chief Investigator. The Trial Management Group (TMG), consisting of the co-investigators, including public and patient representatives, and project staff involved in the day-to-day running of the trial, will meet at least monthly throughout the project. Significant issues arising from management meetings will be referred to the Trial Steering Committee (TSC), Investigators or Funder, as appropriate.

Trial oversight will be provided by a TSC comprising members of the trial leadership team with a majority of independent clinical researchers, as well as a patient representative. The full remit and responsibilities of the TSC will be documented in a charter which will be signed by all members and which is available on the trial website (www.warwick.ac.uk/sinfonia). The Steering Committee will meet annually or more frequently as required and will take responsibility for major decisions, such as a need to change the protocol for any reason, monitoring and supervising the progress of the trial, reviewing relevant information from other sources, and considering recommendations from the DMC.

## Composition of the data monitoring committee, its role and reporting structure {21a}

A data monitoring committee (DMC) will be appointed comprising two independent clinicians with experience in clinical trials and an independent statistician. The roles of the DMC will include monitoring aggregate data and making recommendations to the TSC on whether there are any ethical or safety reasons why the trial should not continue; advising the TSC regarding the release of data and/or information; and considering data emerging from other related studies. The full remit and responsibilities of the DMC will be documented in a charter which will be signed by all members, and which is available on the trial website (www.warwick.ac.uk/sinfonia).

## Adverse event reporting and harms {22}

The potential risk to trial participants is no higher than that of standard care.

As the study involves a population undergoing surgery and general anaesthesia for major surgery, it is anticipated that many participants will experience events which might be considered adverse events (AEs) or serious adverse events (SAEs). Where such events are secondary outcomes or expected features of the perioperative period, e.g. wound dehiscence or infection, paralytic ileus, venous thromboembolism, anastomotic leak, myocardial infarction, these will be captured on case report forms but will not be reported as AEs/SAEs. All AEs and SAEs will be recorded from the time of IMP administration until 24 h thereafter (over five half-lives of the IMPs).

Where an AE/SAE is considered relevant to the trial by clinical or research teams, it is the responsibility of the site investigator to review all relevant medical records and to record relevant information in the CRF and on the SAE form if applicable. This will include assessment of severity and causality. SAEs will be evaluated for duration and intensity according to the National Cancer Institute Common Terminology Criteria for Adverse Events (CTCAE) Version 5.0.

Once received, an independent causality assessment will be undertaken by the CI or delegate. For any SAEs which are suspected to be caused by the trial intervention by either the CI or site clinician, this will be deemed a Serious Adverse Reaction (SAR), and expectedness will be confirmed by the CI or a delegate with reference to the relevant reference safety information (RSI) (the relevant summary of product characteristics for each IMP). SAEs that are deemed to be unexpected and related to the intervention will be deemed Suspected Unexpected Serious Adverse Reactions (SUSARs) and will be notified to the research ethics committees (REC), Medicines and Healthcare Regulatory Authority (MHRA) and sponsor within the relevant deadline. All such events will be reported to the Sponsor, TSC and DMC at their next meetings.

AEs that relate specifically to allergic sensitisation sub-study procedures will be recorded from the time of the allergy clinic appointment until 5 days thereafter. AEs that relate specifically to biological sampling procedures will be recorded from the time of the baseline blood sample until 24 h after the second blood sample has been collected.

## Frequency and plans for auditing trial conduct {23}

A risk-based proportionate approach to monitoring is outlined in a monitoring plan and will be the responsibility of the CTU and sponsor. Monitoring activity will be predominantly central and remote. The trial Risk Assessment and Monitoring Plan will detail the risks identified and controls and mitigation measures in place.

## Plans for communicating important protocol amendments to relevant parties (e.g. trial participants, ethical committees) {25}

All protocol amendments will be coordinated by the SINFONIA trial team based at Warwick CTU and will be subject to approval by the chief investigator, TMG, sponsor, REC and MHRA (if appropriate). Substantial amendments will be subject to additional approval from the TSC, DMC, and funder. Sites will be notified of each amendment by the CTU. The trial registry (ISCTRN) will be updated following each substantial amendment.

## Dissemination plans {31a}

A scientific report will be drafted by the chief investigator and writing committee on behalf of the SINFONIA trial group in accordance with the Consolidated Standards of Reporting Trials (CONSORT) guidelines ([26]). A Publication and Dissemination Plan for SINFONIA will be written and available in the TMF. An inclusive approach to authorship will be used, with trial team members named individually and other participating investigators appropriately acknowledged through group authorship (SINFONIA trial group). All authors will meet the international committee of journal editors’ criteria for authorship.

Patient research partners will help with the production of a plain English summary of trial results, and a video and/or infographic will be produced to aid participants and the public in understanding the trial results. These will be made available to participants and the public via the trial website. Where patients have consented for their email/postal address to be collected, a notification will also be sent to them to advise the results are available.

## Discussion

Despite changes in surgical and anaesthetic practice, major surgery continues to be associated with unacceptably high rates of post-operative pulmonary complications. The use of neuromuscular blocking agents is consistently identified as a risk factor for PPCs, thought to be mediated by inadequate reversal of neuromuscular blockade and weakness of airway and respiratory muscles.

Patients aged over 50 years undergoing major abdominal or thoracic surgery are a large and diverse patient group at elevated baseline risk of PPCs, with a high incidence of co-morbidities such as malignancy, which are associated with additional risk. The inclusion criteria were selected with this prognostic enrichment in view.

Only two reversal agents for neuromuscular blockade are in widespread use in the UK: sugammadex and neostigmine. Sugammadex has obvious advantages, notably faster onset of action leading to more rapid return of neuromuscular function, and this has led to use in an increasing proportion of anaesthetics administered in the UK. However, its clinical effectiveness in preventing adverse patient-centred outcomes is uncertain, and the risk of future allergic reactions is unquantified. Dosing of both drugs is individualised by anaesthetists considering clinical circumstances and the results of neuromuscular monitoring which is deemed essential in national guidelines.

The primary outcome measure, DAH-30, is a composite outcome which reflects patient recovery after major surgery through length of hospital stay, readmission, and death. Lower DAH-30 is associated with age and co-morbidities, but also with the occurrence of complications, including PPCs. It is thus an ideal outcome measure for pragmatic clinical trials such as this.

A strength of the trial is the embedded observational substudy to investigate allergic sensitisation. This will be the largest of its kind to investigate potential sensitisation rates following administration of sugammadex in a routine NHS clinical setting. By combining skin testing with mast cell activation testing, the relative advantages of these techniques will be directly compared for the first time. Collectively, these data will inform our understanding of likely sensitisation rates as usage of sugammadex increases as well as the ideal allergy testing pathway for suspected sugammadex allergy.

Limitations of the trial are largely a reflection of the pragmatic nature of clinical effectiveness trials. The SINFONIA compares two interventions which are both in widespread use, in a real-world context. In clinical practice, the choice of drug, the precise dose and timing, together with the neuromuscular monitoring strategy used to guide therapy and other aspects of concomitant care, are highly variable, largely driven by anaesthetist preference. These preferences are likely to be evident in the trial population, potentially impacting on recruitment to the trial, delivery of trial interventions, and on patient outcomes. These and other relevant factors will be explored in detail in an embedded process evaluation. In particular, the use of quantitative neuromuscular monitoring, although deemed essential according to national guidelines, is not universal. While we highlight the importance of providing high-quality concomitant care including adherence to these guidelines in the trial, the trial aim is to evaluate the two drugs in the context of usual clinical care: to mandate a specific neuromuscular monitoring strategy would be to undermine the pragmatic nature of the trial and reduce external validity.

In conclusion, the SINFONIA trial addresses an important question for anaesthetists and for patients undergoing major abdominal and thoracic surgery. This pragmatic clinical effectiveness trial will provide robust evidence as to the risks and benefits of sugammadex compared with neostigmine on patient-centred outcomes such as DAH-30, as well as on cost effectiveness and the incidence of allergic sensitisation.

## Trial status

The first patient was enrolled to the SINFONIA trial on 15th February 2023, and the 12-month internal pilot phase was completed on 30th November 2023. Trial recruitment continued seamlessly into the main phase and is anticipated to complete by the end of 2025.

## Supplementary Information

Additional file 1. Examples of eligible major surgical procedures.

## Data Availability

The final trial dataset will be maintained at the University of Warwick Clinical Trials Unit. Access to the dataset will be subject to approval from the trial management group and may be requested through contact with the chief investigator following publication of trial results.

## Abbreviations

AE: Adverse event
AUC: Area under the curve
BHSCT: Belfast Health and Social Care Trust
CDMS: Clinical Data Management System
CHEERS: Consolidated Health Economic Evaluation Reporting Standards
CI: Chief Investigator
CONSORT: Consolidated Standards of Reporting Trials
CRF: Case Report Form
CTCAE: Common Terminology Criteria for Adverse Events
DAH-30: Days alive and out of hospital at 30 days
DMC: Data Monitoring Committee
EQ-5D-5L: EuroQoL 5-Dimension 5-Level
GCP: Good xlinical practice
IgE: Immunoglobulin E
IMP: Investigational medicinal product
ISRCTN: International Standard Randomised Controlled Trial Number
IVRS: Interactive Voice Response System
mg: Milligrams
MHRA: Medicines and Healthcare products Regulatory Agency
NHS: National Health Service
NICE: National Institute for Health and Care Excellence
NIHR: National Institute for Health and Care Research
NMBA: Neuromuscular blocking agent
PE: Process evaluation
PI: Principal Investigator
PPC: Post-operative pulmonary complications
QoR-15: Quality of recovery-15
QALY: Quality adjusted life year
REC: Research Ethics Committee
R&D: Research and Development
RSI: Reference Safety Information
SAE: Serious adverse event
SAIL: Secure Anonymised Information Linkage
SAP: Statistical analysis plan
SD: Standard deviation
SINFONIA: Sugammadex for prevention of post-operative pulmonary complications
SPIRIT: Standard Protocol Items: Recommendations for Interventional Trials
SOP: Standard operating procedure
SUSAR: Suspected unexpected serious adverse reactions
TMG: Trial Management Group
TSC: Trial Steering Committee
UK: United Kingdom
WCTU: Warwick Clinical Trials Unit
