# Supplementary material for: Sugammadex or Neostigmine for prevention of post-operative pulmonary complications after major abdominal or thoracic surgery: study protocol for the SINFONIA (Sugammadex for preventioN oF pOst-operative pulmonary complIcAtions) randomised controlled superiority trial
Source: Trials. 2025 Aug 28;26:319. doi: 10.1186/s13063-025-08987-4 (PMC12392639; doi:10.1186/s13063-025-08987-4)
Supplement: Supplementary file 1 — Additional file 1. Examples of eligible major surgical procedures. [file 13063_2025_8987_MOESM1_ESM.docx]

# **APPENDIX: EXAMPLES OF ELIGIBLE MAJOR SURGICAL PROCEDURES**

Major surgical procedures are expected to last more than 90 minutes, with significant risk of tissue injury and complications, and include the following examples. This list is not exhaustive but simply intended to provide a guide for researchers in assessing participant eligibility. Some procedures (e.g. laparoscopic hysterectomy) can be of variable magnitude, and eligibility will depend on a detailed knowledge of local practice and individual patient factors.

Gastrointestinal:

Gastrectomy

Oesophagectomy

Fundoplication

Cardiomyotomy

Pancreatectomy

Pancreatic transplant

Bowel resection

Hartmann’s procedure

Splenectomy

Adrenalectomy

Hepatic resection

Liver transplant

Component separation repair

Exploratory laparotomy

Repair of perforated ulcer

Gynaecological:

Total abdominal hysterectomy

Cytoreductive surgery

Pelvic exenteration

Thoracic:

Diaphragm repair

Pneumonectomy

VATS/open lobectomy

Pleurectomy

Thymectomy

Urological:

Cystectomy

Nephrectomy

Radical prostatectomy

Renal transplant

Repair of vesico-colic fistula

Vascular:

Aorto-femoral bypass

Axillo-femoral bypass

Abdominal aortic aneurysm repair

Thoracic aortic aneurysm repair

the following are eXAMPLES OF SURGERY WHICH IS NOT CONSIDERED MAJOR AND THEREFORE **NOT ELIGIBLE** FOR INCLUSION:

Laparoscopic cholecystectomy, inguinal hernia repair, appendicectomy, vaginal hysterectomy, diagnostic mediastinoscopy.
